# Comment on epinephrine during resuscitation of traumatic cardiac arrest and increased mortality: a post hoc analysis of prospective observational study

**DOI:** 10.1186/s13049-019-0686-3

**Published:** 2019-11-27

**Authors:** Makoto Aoki, Toshikazu Abe

**Affiliations:** 1 Department of Emergency Medicine, Gunma University Graduate School of Medicine, Maebashi, Japan; 2 Department of General Medicine, Juntendo University, Tokyo, Japan

**Keywords:** Epinephrine, Traumatic cardiac arrest, Biases, Vasopressin

## Abstract

The aim of this Letter to the Editor was to report some important biases in a recently published Article. We agreed with the notion by Yamamoto et al. that the effects of epinephrine regarding was limited without hemostasis, however, this study had major limitations such as no information on etiology of traumatic cardiac arrest (hemorrhagic or on non-hemorrhagic) and on hemostatic treatment. The results of this study should be interpreted with caution and further analysis is necessary. Finally, we commented on the necessity of future study regarding another vasopressor (ie; vasopressin) on traumatic cardiac arrest based on current evidence.

Dear Editor.

We read with interest the article of Yamamoto, et al., who reported that epinephrine administration during in-hospital resuscitation of traumatic cardiac arrest was associated with increased mortality [1]. Yamamoto et al. [1] performed post-hoc data analysis on a prospective, multicentre, observational study (SOS-KANTO 2012) consisting of patients who suffered out-of-hospital cardiac arrest and were transported to 67 emergency hospitals by emergency medical services personnel. We agree with the notion by Yamamoto et al. that the effects of epinephrine regarding spontaneous circulation would not persist without hemostasis, and our previous investigation demonstrated that prehospital epinephrine administration was associated with increased temporal return of spontaneous circulation but was not associated with survival [2]. The study of Yamamoto et al. [1] demonstrated that administration of epinephrine was associated with increased mortality even in in-hospital situations where prompt hemostatic treatment could be deployed.

However, we have to interpret the findings of the study of Yamamoto et al. [1] while considering some major limitations. The most important limitation is that the main etiology of traumatic cardiac arrest in the subjects was not known in this study, that is, whether the etiology was hemorrhagic or non-hemorrhagic. Lin, et al. [3] showed that the effect of epinephrine on traumatic cardiac arrest in children differed depending on whether the etiology was hemorrhagic or non-hemorrhagic. Additionally, the details of hemostatic treatment such as emergency thoracotomy, resuscitative endovascular balloon occlusion of the aorta and time to treatment were not entered in the SOS-KANTO 2012 database. In-hospital procedures vary among hospitals and this bias could have affected the results.

Currently, vasopressin is the only vasopressor that has the possibility of improving the outcome of trauma patients [4]. A recent randomized controlled trial showed that administration of low-dose arginine vasopressin to hemorrhagic shock patients decreased blood product requirements [4]. No study has investigated the effect of administration of vasopressin on traumatic cardiac arrest; therefore, further trials are needed.

## Data Availability

Data sharing not applicable to this article as no datasets were generated or analyzed during the current work.
